# Misdiagnosed Pityriasis Rubra Pilaris Successfully Managed With Isotretinoin: A Case Series

**DOI:** 10.7759/cureus.38657

**Published:** 2023-05-07

**Authors:** Nayla Al Khalifa, Manal Alsabbagh, Mazen Raees, Eman Aljufairi

**Affiliations:** 1 Dermatology, King Hamad University Hospital, Busaiteen, BHR; 2 Pathology, King Hamad University Hospital, Busaiteen, BHR

**Keywords:** isotretinoin, keratinization, atopic dermatitis, papulosquamous disorders, pityriasis rubra pilaris

## Abstract

Being a rare inflammatory, hyperproliferative dermatosis, diagnosing pityriasis rubra pilaris (PRP) can be a challenge to many clinicians. Our case reports aim to demonstrate that PRP is frequently diagnosed and managed as atopic dermatitis (AD) and that distinguishing features on dermoscopy, and biopsy can help diagnose this rare disease. The study also aims to show that PRP can be successfully treated with Isotretinoin.

Our case series aims to describe two cases of PRP, initially diagnosed as AD and managed with topical corticosteroids. Being the first case series in the literature to describe the incidences of diagnosing PRP as AD portrays the significance of utilizing dermoscopy and biopsy as tools to confirm this diagnosis for appropriate management.

Although PRP is a rare diagnosis, dermoscopy and biopsy can help confirm the disease. Management with isotretinoin will most likely have successful outcomes in those patients.

## Introduction

Pityriasis rubra pilaris (PRP) is a rare papulosquamous disorder with an incidence rate ranging between one in 5000 dermatology visits in the UK [1] and one in 50,000 visits in India [2]. It targets equal numbers of males and females [1]. PRP typically presents with follicular hyperkeratotic papules and orange erythroderma, with islands of sparing; palmoplantar keratoderma is commonly seen [3].

The pathophysiology remains unclear, nevertheless, studies reported that there may be an association with CARD 14 (caspase recruitment domain-containing protein 14) mutations, activating the Th17 pathway, as well as infections, autoimmune, and neoplastic triggers [4,5]. Wang et al. highlighted the possibility of vitamin A's role in the pathogenesis as discussed later [3].

Although histology remains the gold standard for diagnosing PRP, dermoscopy has been a useful tool in clinically distinguishing PRP from other diagnoses, such as psoriasis and atopic dermatitis (AD) [6,7]. Histopathology often demonstrates psoriasiform hyperplasia and alternating orthokeratosis and parakeratosis, in vertical and horizontal planes [6,7].

We are presenting two cases of PRP in which we aimed to discuss the utility of dermoscopy and biopsy in distinguishing PRP from other diagnoses, mainly AD.

## Case presentation

Case 1

**Figure 1:**
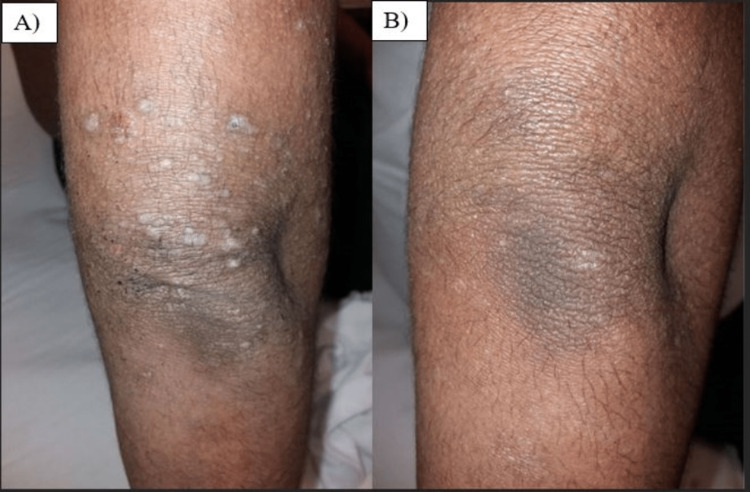
A) Lichenified plaques and excoriations on the right elbow; B) Residual hyperpigmentation in the right elbow, after treatment with isotretinoin

**Figure 2:**
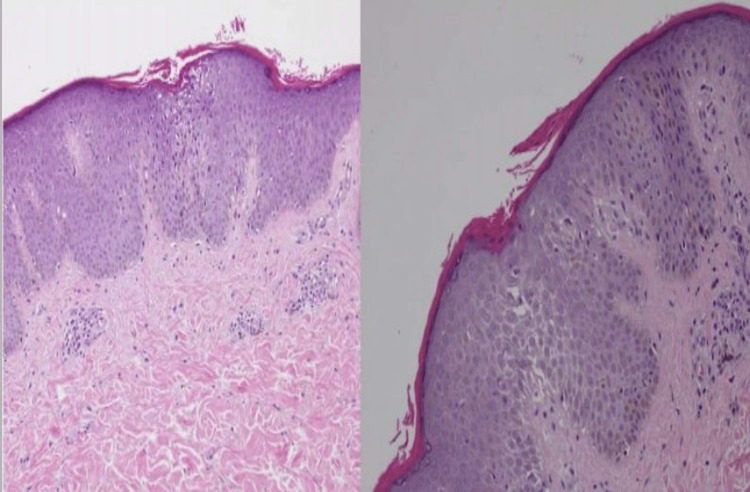
Skin biopsy shows mild spongiosis and lamellar orthokeratosis with patchy hyperkeratosis. A mild perivascular lymphocytic infiltrate is also noted. (Hematoxylin and eosin stain, original magnification x100 and x200)

A 16-year-old male presented to the dermatology clinic with itchy eczematous skin changes involving the face, trunk, and upper and lower extremities. He was previously diagnosed with generalized AD and managed with topical corticosteroid mixtures along with moisturizers on two consecutive visits, which only gave him partial relief. Previous investigations showed an elevated immunoglobulin E (IgE) of 712 international units per milliliter (IU/mL). On examination, he had generalized follicular hyperkeratosis with islands of sparing and lichenified plaques on his elbows (Figure 1A). The clinical presentation raised suspicion of PRP, so a skin biopsy was performed and the histology confirmed the diagnosis of PRP (Figure 2). He was started on once-daily 20 milligrams (mg) isotretinoin capsules. After two weeks of treatment, the patient confirmed an improvement of approximately 30% in the skin texture, however, still experienced occasional itching. After six weeks, the patient noted significant improvement in overall skin condition and had no complaints of itchiness (Figure 1B).

Case 2

**Figure 3:**
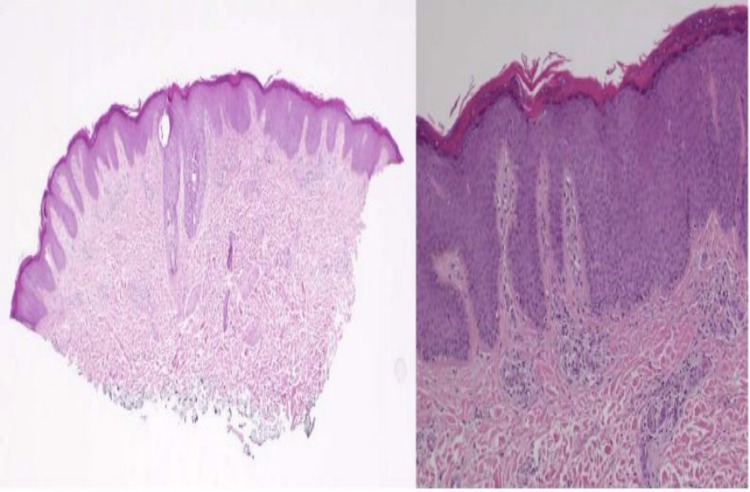
Skin biopsy shows lamellar orthokeratosis, hypergranulosis, and broad elongated rete ridges. A mild superficial perivascular lymphocytic infiltrate is noted. (Hematoxylin and eosin stain, original magnification x20 and x100)

A 21-year-old male patient, diagnosed with AD, presented to the dermatology clinic, in January 2019, with complaints of a flare of his dermatitis and acne. On examination, he had severe generalized skin xerosis, follicular keratoses, and lichenified dermatitis patches on the elbows and knees. Furthermore, multiple nodulocystic papules, pustules, and scars were present on the face. At that time, he was assessed as having severe AD and moderate to severe acne, and was given fluticasone propionate 0.05% ointment, topical clindamycin phosphate 1% solution, adapalene 0.1% gel, emollients, anti-histamine, and once-daily doxycycline 100 mg capsules, and was planned to start isotretinoin. Subsequently, in July 2021, he presented for follow-up with new complaints - in addition to severe dermatitis and acne. He was having right cheek swelling and multiple scalp swellings as well as boils in the axillae. He was reviewed by an ENT consultant, and a computed tomography (CT) scan was done and revealed vascular malformation of the right cheek, with incidental findings of thyroglossal duct cyst and left Sylvian fissure arachnoid cyst. In the next follow-up in March 2022, thorough investigations were carried out. Pelvis, neck, and groin ultrasound (US) showed multiple reactive lymphadenopathies, some being greater than 20 millimeters (mm). IgE was greater than 1200 IU/mL, yet IgA, IgM, and IgG were within normal limits. Peripheral smear showed eosinophilia. Syphilis, human immunodeficiency virus (HIV), and autoimmune nuclear antigens (ANA) screens were negative. Chest X-ray, other routine laboratory investigations, and abdomen US were normal. After these investigations, differential diagnosis was adjusted to acne conglobata with atopy, and the patient was managed with a mixture of betamethasone valerate 0.1% and fusidic acid 2% cream. The consequent visit showed pus-draining nodules in the axilla, and a pus swab revealed *Staphylococcus aureus*. As his dermatitis was not improving with topical steroids, a skin biopsy was done and it showed classical findings of PRP (Figure 3).

In April 2022, the patient was started on once-daily 10 mg isotretinoin oral capsules, with a target dose of 4800 mg. Subsequently, the cystic lesion on the scalp became smaller and not erythematous and showed hair growth. The patient’s liver function tests and lipid profile were observed monthly, which were all normal. His dermatitis lesions subsided completely after three months after initiating isotretinoin. However, he continued developing on and off crusted papules and nodules on the face. Thus, a tapering dose of 20 mg prednisolone tablets was added to the regimen in September 2022. Prednisolone was tapered over seven weeks. Thereafter, in December 2022, an examination of the body showed residual dermatitic patches on the left arm. With regards to acne conglobata and hidradenitis suppurativa, further visits showed no improvement of the lesions on the face or axilla, which encouraged further therapies to be attempted.

## Discussion

Our results suggest that failure to control chronic AD lesions should prompt further dermoscopic assessment to search for PRP features and should also encourage an attempt of biopsy to confirm possible a diagnosis of PRP and rule out other possible differential diagnoses such as cutaneous T-cell lymphoma.

Although PRP usually presents with hyperkeratotic follicular papules, palmoplantar keratoderma, and red-orange scaling plaques involving the scalp, trunk, and limbs, with well-demarcated “islands of sparing” [6], our case series show that both PRP and AD can present with lichenified dermatitis or eczematous patches, as usually seen in AD [8]. The most frequent dermoscopic criteria of PRP are the presence of central hair, follicular plugs, and perifollicular yellow/orange halos, whilst psoriasis is characterized by regularly distributed dotted vessels and white scales [1].

No standard protocol is available for treating PRP, as no randomized control trials have been attempted yet [9]. However, PRP is initially treated via emollients and keratolytic agents, such as urea 40% cream, topical corticosteroids, and vitamin D analogs [7,10]. Moderate to severe disease, or failure to respond to potent topical corticosteroids, warrants the use of systemic therapy [10]. Clinicians prefer to start such patients on isotretinoin, with a recommended dose of 1mg/kg/day [7]. Methotrexate (MTX) is a second-line treatment, which can be used alone or in combination with isotretinoin, despite the increased risk of hepatotoxicity, pancytopenia, pneumonitis, and teratogenicity [7]. More refractory cases can be managed with azathioprine and cyclosporin [7,9]. A recent case series demonstrated a 90.9% response rate to MTX use alone, with complete clearing of lesions in 40.9% of patients, and was only associated with mild side effects in only 12.9% of patients [7,9].

Biologic therapy, such as anti-tumor necrosis factor (anti-TNF) alpha agents, interleukin-17 (IL-17), and interleukin IL-23 inhibitors, have recently emerged in successfully treating refractory PRP cases [7,9,11]. A recent systematic review identified that biologic treatment was successful in 81.1% of the patients when used as monotherapy or combination therapy with existing treatments [7,11]. Recent case reports demonstrated the effectiveness of the combination of ustekinumab, an IL-12 and IL-23 inhibitor, with acitretin, in clearing keratoderma within the classic adult, pediatric, and refractory PRP [5,10]. Another systematic review demonstrated that infliximab was associated with a more rapid response than etanercept. On the other hand, ustekinumab monotherapy, or in combination with other regimens, has been proven to give long-term control in treating adult and juvenile PRP [5,10]. A few case reports have also shown clearance of palmoplantar keratoderma in types 1 and 2 PRP within two weeks of using secukinumab [9]. Use of apremilast, extracorporeal photochemotherapy in erythrodermic PRP, and intravenous immunoglobulin have also been reported [7,9].

## Conclusions

PRP remains a rare cutaneous keratinization disorder, which can often be misdiagnosed as AD, psoriasis, follicular eczema, follicular ichthyosis, generalized hypersensitivity reaction, T-cell lymphoma, and lichen planopilaris. The most common misdiagnoses include psoriasis, contact dermatitis, and eczema or spongiotic dermatitis.

Our case series suggests that misdiagnosing PRP is relatively common and that clinicians should utilize dermoscopy and histopathology to confirm PRP in the treatment of recalcitrant cases of AD. This is due to the absence of dotted vessels and white scales on dermoscopy, in the presence of central hair, follicular plugs, and perifollicular yellow/orange halos suggests a diagnosis of PRP; and histology can confirm this diagnosis. Hence, further large-scale studies can confirm that histology and dermoscopy are needed to distinguish PRP from AD.

Although no standardized treatment protocols have yet been established, our case series shows that treatment with isotretinoin in moderate PRP can be successful.
